# Individuals’ contribution to household energy savings: The role of big-two personality traits

**DOI:** 10.1016/j.heliyon.2024.e25922

**Published:** 2024-02-10

**Authors:** Md Shahin, Milad Ghasri, Alireza Abbasi

**Affiliations:** a School of Engineering & Technology, University of New South Wales, Australia; b Department of Disaster Resilience and Engineering, Patuakhali Science and Technology University, Bangladesh; c School of Systems & Computing, University of New South Wales, Australia

**Keywords:** Household energy consumption, Stability, Plasticity, Personality traits, Structural equation modelling

## Abstract

Household energy consumption (HEC) is one of the major contributors to global emissions, making it a critical area for addressing sustainability challenges. The impact of personality traits on human behaviour is significant in shaping HEC patterns, and therefore, have important implications for sustainability policies. This study aims to investigate role of biologically predicted big-two personality traits (i.e., stability and plasticity), a higher order solution to five-factor traits and orthogonal traits, on HEC. To that end, a structural equation model is developed using a national household survey in Australia. The performance of the model is benchmarked against a five-factor (i.e., agreeableness, consciousness, emotional stability, extraversion and openness) personality trait model. The performance of the models is measured using six goodness-of-fit indices, all of which show a superior performance in the big-two traits model. The results indicate that a higher score in stability poses energy-intensive behaviour, while a higher plasticity score poses energy-saving behaviours. The plasticity trait is linked to environmentally friendly behaviours, while the stability trait is associated with environmentally unfavourable behavioural practices. The effects of socioeconomic status on HEC are mediated by stability and plasticity to identify those who are more likely to change their energy consumption habits as the target group for policy intervention. This study can assist policy makers to determine energy-intensive and energy-saving behaviours from the big-two traits, and to develop more effective and targeted sustainability policies that can help in reducing HEC and promote sustainable living in societies.

## Highlights

• Stability and plasticity traits strongly influence individuals' household energy consumption behaviours.
• Stability relates to higher household energy consumption.
• Plasticity trait correlates negatively with household's energy consumption.
• Individuals with higher plasticity have a greater likelihood of choosing sustainable energy consumption options.
• Higher stability maintains consistent behaviour , fostering predictability for sustainable energy consumption practices.

## 1 Introduction

Global energy consumption (EC) results in global warming, climate change and frequent natural disasters [1]. The household sector accounts for 25% of global EC [2], and contributes 17% of global ${CO}_{2}$ emissions [3]. A recent study [4] reports per capita emissions linked to energy use in 2015 in Peru was 305 kg ${CO}_{2}$ eq. However, HEC is a key component in the endeavour for rapid emission reduction. Achieving net zero emissions (NZE) by 2050 requires a 55% reduction in emissions from EC by 2030 [5]. To do so, much attention is paid to the technical improvement of buildings [[6], [7], [8]], green building features [9], and retrofitting [8,10]. Low-carbon domestic house building design significantly reduces [11], where technological improvement can cut 30% of residential energy use [12,13]. Beyond technical factors, residents’ behaviour, including building characteristics and demographics, influences HEC thus affecting environmental sustainability.

Building characteristics, e.g., house value, rooms, and age have significant effects on HEC [14,15]. Research on the role of socioeconomic status (SES) finds income [16,17], age [18], and gender [19] to affect HEC. Psychological constructs, including beliefs, perceptions, attitudes, and personality traits, also play a significant role in HEC [16,20]. Residents’ behaviour can efficiently reduce up to 25% of EC and greenhouse gas (GHG) emissions [21]. The five personality traits of agreeableness, consciousness, neuroticism, extraversion, and openness, which are referred to as the heritable characteristic patterns of thoughts, feelings as well as behaviours, have significant but sometimes contradicting impacts on the green behaviour (GB) context (i.e., pro-environmental and energy-saving behaviour). While some studies have reported a positive impact on pro-environmental [22,23], and energy-saving [24,25] behaviours, others have identified a negative relationship between these traits and such behaviours [26,27].

Studies exploring the association between big-five traits and HEC have yielded mixed findings. It is important to note that the big-five traits do not represent the broadest level of personality due to non-orthogonal nature and obliqueness [[28], [29], [30]] and intercorrelated [[31], [32], [33]]. While some research proposes two orthogonal traits as a higher-order solution to the big-five traits [29,30,34,35], the big-two traits have emerged as a simpler, higher-order solution [35] have identified these two traits as stability and plasticity, which are meta-traits of the human brain system [35,36]. The dopaminergic system regulates plasticity, while stability is governed by the ascending rostral serotonergic system [35]. Therefore, comprehensive knowledge of the relationship between big-two traits and EC behaviour can help policymakers tailor their policies to better resonate with different individuals. By identifying individuals with different preferences and motivations, policymakers can innovate approaches that are more inclusive, effective, and sustainable. Unlike the big-five traits which have been investigated in GB context, the big-two traits have not received adequate attention. Previous studies show consistent effects from the big-two traits in other contexts e.g., social conformity [35], creativity [30,37,38], social valuation [36], and mindfulness [39], and this consistency is expected to extend to EC as well.

Understanding how stability and plasticity affect HEC can facilitate devising more effective policies for reducing HEC and greenhouse gas emissions. While some studies, e.g. Ref. [40], suggest a correlation between personality traits and SES, others argue they are independent, e.g., Ref. [41]. To identify the target groups while developing a numerical tool to support policy, it is necessary to link personality traits and SES. The proposed framework will explore the possible relationship between SES, big-two traits, and HEC using data from the national household survey of Household, Income and Labour Dynamics in Australia (HILDA). Structural equation modelling (SEM) will be used to quantify the effects of big-two traits and SES on HEC as it is a reliable tool for behavioural modelling.

The paper is structured as follows: Section 2 reviews literature on GB, personality traits (big-five and big-two scales), and policy aspects of HEC. Section 3 outlines the study's methodology, including data processing and analysis methods. Section 4 presents results, followed by a comprehensive discussion of the big-two traits, SES, and HEC relationship in Section 5, with policy implications and study limitations. Finally, Section 6 summarises the study's highlights.

## 2 Literature review

Recent research has focused on human behaviour in energy-related studies, particularly on GBs at both aggregated and individual levels, considering attitudes, social norms and perceived behavioural control [42,43]. The big-five traits have been extensively studied in relation to GBs, but their direct relationship with HEC remains unstudied. The effects of personality traits on green behaviour are mixed, indicating their potential role in policy development to reduce HEC. Personality traits reflect how one makes decisions [44]. The big-five traits are considered the highest level of personality traits in psychology [28,[31], [32], [33]]. However, studies have shown that they are regularly correlated. Additionally, the big-two scale is a standard higher-order scale summarising the big-five scale in itself.

### 2.1 The big-five traits and green behaviour

Personality traits describe the individual differences and uniqueness [45]. Allport defined personality is “the dynamic organization within the individual of those psychological systems that determine his unique adjustments to his environment” [46]. Personality traits as the basic requirement of human heterogeneity lead to distinct environmentally conscious behaviour [47]. Big-five traits have been widely studied in the context of GB [24,26,48]. There is no direct study of the big-five impact on HEC. Therefore, the brief literature review focuses on the link of personality traits and GB.

Agreeableness is the degree to which a person shows compassion or antagonism in their thoughts, emotions and actions [45]. Highly agreeable people are typically warm, caring, empathetic, cooperative, and trustworthy [30,49]. The identified effects of agreeableness on GB are mixed. While some studies have found a positive relationship between [25,26,50], indicating that agreeable people are more likely to follow GBs due to their empathetic nature and desire to improve the lives of others, others [23,49] suggest a negative impact as agreeable individuals may avoid risk-taking behaviours that could affect their relationship with GB. Some studies [48,51,52] find insignificant relationships and suggest that good-natured and sociable individuals may be less interested in environmental issues as they consider others' contexts.

Conscientiousness measures an individual's reliability, organisation, persistence, and motivation in goal-oriented behaviour [49]. Most previous studies report a positive association between conscientiousness and GB. Individuals with higher conscientiousness have a higher level of empathy and self-transcendence motivating them to adhere to proper environmental actions [22,26,50], and controllability towards energy conservation [25]. Nonetheless, the relationship is found insignificant in some studies [23,48,53] and occasionally a negative relationship is reported [27]. They argue that individuals with higher conscientious have a desire to “do the right thing” and they are well socialised which can be associated with the performance of environmentally conscious behaviours.

“The emotional stability (opposite to Neuroticism) characterises someone as calm, self-confident and secure” [49]. Neuroticism is characterised by a tendency to experience psychological distress and maladaptive coping mechanisms [45]. The relationship between emotional stability and GB is mixed in past studies. A few studies [51,54] found insignificant effects and they argue that certain environmental engagement (e.g., preservation attitudes) may be associated with widespread inclinations to experience high levels of anxiety and emotional volatility [55]. Many of the past studies [22,26,50] show a positive effect of neuroticism on pro-environmental behaviours and they argue those with higher levels of neuroticism may have greater anxiety about the consequences of environmental deterioration [22,50]. Some studies [25,26,53] reported a negative relationship between neuroticism and environmental engagement, which they explain as being due to the adverse affective characteristics associated with higher neuroticism such as sensitivity, anxiousness, and insecurity [53].

Extraversion measures the level of activity, demand for stimulation, interpersonal interaction, and joyfulness capacity [45]. While studies on the relationship between extraversion and GB have produced mixed results, most studies have found a positive association, suggesting that individuals with higher extraversion are more likely to engage in eco-civic and sustainable outdoor activities [25,48,52,56]. Few past studies [26,27] report a negative relationship without providing a clear explanation. Several studies [22,23,50,53,57] show insignificant impacts. They argue that people are subjected to fewer judgments and expectations from others, and they are not required to take extra steps to engage in social activities at home [53].

The “openness to experience” dimension involves a range of interests and fascination towards novelty. Highly open individuals are creative, curious, and artistically sensitive [49]. The identified effect of openness on GBs is positive. Studies [22,23,50,52,53,56] have reported a positive relationship and they argue those with higher openness tend to be more open to new ideas and experiences, and highly connected to nature [57]. Open individuals have abstract thinking [54], high levels of aesthetic appreciation, creativity, and inquisitiveness [48], making them adaptable to changing contexts [49], and flexible in both behaviour and thought [30]. However, very few studies [26,53] find an insignificant relationship, which can be because of small sample sizes.

### 2.2 The big-two traits and green behaviour

The cybernetic big-five theory constructs two higher-order personality meta-traits of stability and plasticity [39,58,59], which are theorised as fundamental needs for biological survival [60]. Deyoung et al. [35] find that the big-two traits appear to be inherited very early in ontogeny, and that a more basic, biologically predicated, interpretation of the big-two might be justified.

Stability consists of shared variance of emotional stability, agreeableness, and conscientiousness. Emotional stability refers to relative freedom from adverse effects and behavioural withdrawal [31,[61], [62], [63]]. Agreeableness maintains the secured social relationships [31,64]. Conscientiousness maintains motivational stability through organised efforts [31]. These traits are interconnected and work together to maintain stability in social, emotional, and motivational domains. The cybernetic systems control goal-oriented behaviour when encountering disturbing influence by stability meta-trait [39]. Individuals with higher stability are characterised by a lower capacity for adapting to varied circumstances, and reconceptualisation to adapt to new situations. They often seek to avoid distress, uncertainty, and hostility and may exhibit a fear of the unknown and an aversion to novelty [30,35].

Plasticity trait consists of shared variance of extraversion and openness to experience traits. Extraversion is connected to sociability [65,66]), positive emotion, incentive reward sensitivity, approach behaviour and craving novelty [31,61,[67], [68], [69]]. Openness reflects the tendency to broaden experiences, curiosity, creativity, flexibility, and willingness to challenge social norms [70]. Plasticity entails a propensity towards curiosity, adapting to novel situations, flexibility, challenging societal conventions, seeking out stimulating experiences, and having the inclination to experience positive affectivities [30]. The central dopaminergic system mediates the approach behaviour, positive aspect, and incentive reward sensitivity [71,72], and is related to the responses to novelty [72,73]. The plasticity is the technique by which the cybernetic systems approach and explore the unfamiliar, either cognitively (i.e., openness) or behaviourally (i.e., extraversion) [39].

There is no study on the big-two traits in the context of GB, energy saving and EC. This is while the relationship between the big-two traits and many other social and individual behaviours, e.g., social conformity [35], social valuation [36], social network use [74], creativity [30,37,75] and mindfulness [39] are well-explored. In all these applications, the identified relationships are consistent. Little is known about the relationship between the big-two and HEC, and it is expected that a reliable and quantitative model that connects the two will support policy makers to develop more targeted energy related interventions to meet NZE target.

### 2.3 Policy and HEC

Energy-saving policies and programs aim to enhance energy efficiency and reduce EC and consequently GHG emissions [12]. Most of these policies focus on improving energy efficiency in buildings and appliances [76], energy structure change [77], and financial incentives for green building [78,79]. For instance, the European Commission (2013) has set a nearly zero-energy building standard for NZE. The Intergovernmental Panel on Climate Change has suggested building efficiency improvement as most cost-effective climate change mitigation approach in the construction sector [80]. Previous studies show policies that incorporate current technologies can contribute to a 30% reduction in HEC [12,78].

Besides building retrofitting, policies promoting sustainable behavioural change are necessary to achieve target consumption levels [8]. Behavioural changes can contribute to an additional 20% reduction in EC. The widely known stimulus that is commonly used by policymakers to adjust consumption is price. It is not surprising to see as electricity prices increase EC decreases [81]. However, not all the policies devised based on price are successful and they can raise fairness issues for low-income groups. Success depends on various factors, including consumers' SES, environmental awareness, demographics, lifestyle, and behaviour, all of which can affect EC and policy outcomes [12]. It also comprises other factors including how well we know the consumers, their expectations, and their behaviour. In the behavioural process, personality traits have a significant role in HEC, which need to be adopted in the devising of energy regulation interventions. For HEC estimation and intervention strategy development, it requires a comprehensive insight into the motivations that influence HEC.

Reducing EC to achieve the NZE target requires both technological improvement and behavioural shifts. Individual's GB plays a significant role in reducing HEC, which could be incorporated into the strategical development of energy policies. Devising effective policies in promoting GBs requires a good understanding of personality traits. The role of personality traits on EC behaviour from the lenses of policy making is not sufficiently explored. This study will bridge this gap by exploring the impacts of the big-two traits on HEC, and the relationship between personality traits and SES. SES will help in identify the personality traits and therefore, policy maker can capture individuals globally to develop more targeted interventions.

## 3 Data and methodology

### 3.1 Research data

This study uses the HILDA dataset to investigate the impact of stability and plasticity on HEC. The HILDA survey commenced in 2001, is a nationally representative longitudinal study of Australian households. The HILDA study follows members of a nationally representative sample of Australian households on an annual basis [82]. The HILDA survey gathers data on family life and labour market trends and individuals' well-being. The HILDA follows the lives of more than 17,000 Australians. This study utilises the 2017 wave of HILDA data as it is the latest to include personality trait data, collected every five years. HILDA encompasses 1400 variables by covering various aspects of residents' lives, including socioeconomic and demographic characteristics, household relationships, income, occupation, health, education, building characteristics, personality traits, EC, and contextual factors.

In HILDA, data collection occurs on an annual basis through a combination of interviews and the distribution of self-administered paper forms, a self-completion questionnaire (SCQ). The interviews consist of two main parts: one part is conducted with a single individual within the household and typically takes around 12–13 min. The second part, which is given to every household member who is 15 years of age or older, typically takes 35 min to complete [82]. Most interviews are face-to-face and typically take place in the respondent's home. However, the telephone is employed when respondents demonstrate a strong desire for a telephone interview and are located outside the interviewer network, even if it is the last option. Over the past few years, telephone interviews have made up around 8–10% of all interviews [82]. All persons completing a personal interview are given the SCQ, 20-page form. Frequently, it is finished and taken when the interview is taking place. However, when this isn't feasible, our interviewers are committed to making a follow-up visit to collect the completed forms from every household. For telephone respondents, the SCQ is conveniently administered via mail. Approximately 95% of the interviews are conducted between August and November, while the data collection period spans late July to early February the following year [82,83]. The data collection for Wave-17 was started on July 25, 2017 and finished on February 4, 2018. Regarding the scope of research with HILDA survey data, it provides policy-makers with unique insights about Australia, enabling them to make informed decisions across a range of policy areas, including health, education, economic, and social services.

HEC is collected as the annual expenditure on the electricity, gas, and other heating fuel of a household. Regarding Big-five personality traits, participants were asked about their personality character traits using a questionnaire of a 36-item inventory in HILDA. This method was derived from Ref. [84] trait descriptive adjectives approach, which builds upon [85] method. Both approaches assume a 5-factor structure, as commonly accepted in the literature. It's noteworthy that not all 36 items contribute to the five scales representing Agreeableness, Conscientiousness, Emotional Stability, Extraversion, and Openness to Experience. To refine these scales, a two-step process was implemented [86]. Initially, pre-analysis reliability testing of ex-ante scales was conducted, leading to the exclusion of items with an item total correlation below 0.3. Subsequently, a principal components analysis, aiming for a five-factor solution, was performed. Only items meeting specific criteria, such as having the highest factor loading on the anticipated factor, exceeding 0.4, and surpassing the second highest factor loading by at least 0.1, were retained. A slightly different approach, detailed in Ref. [87], yielded identical outcomes in deriving these scales. The resulting scales represent the average scores of the respective personality character items. Agreeableness is composed of cooperative, kind, sympathetic, and warm facets, while Consciousness includes disorganised, efficient, inefficient, orderly, sloppy, and systematic facets. Emotional stability includes envious, fretful, jealous, moody, temperamental, and touchy. Extraversion consists of bashful, extroverted, lively, quiet, shy, and talkative. The facets of openness include complex, creative, deep, imaginative, intellectual, and philosophical. These personality items are measured using a seven-point Likert scale where 1 indicates “doesn't describe me at all”, and 7 represents “describe me very well”.

The adoption of higher-order big-two personality traits is widely endorsed in the existing literature, as emphasized by Refs. [29,34,35], and. In line with this recommendation, our study strategically utilized stability (comprising the shared variance of agreeableness, conscientiousness, and emotional stability) and plasticity (comprising the shared variance of extraversion and openness to experience) as the big-two traits. This framework, originally developed by Deyoung et al. [35], has been successfully applied across various social contexts, as thoroughly discussed in Section 2.2. The incorporation of these big-two traits serves as a robust foundation for our study, aligning with established research and providing a comprehensive framework for our analyses.

### 3.2 Data preparation

The data preparation includes data sorting, treating missing values and outliers, and testing the predictability power of variables on the target variable. The selected variables included those related to HEC, personality traits, SES attributes, and building characteristics. To investigate the personality traits, this study excludes individuals under 15 years old and captures couples from each household. From the selected couple, a representative member from each household is chosen based on the highest education level. This is because education is a critical component for acquiring knowledge, awareness, skill training and behavioural change, and it promotes cleaner energy consumption [88,89]. Education stands as a crucial factor influencing the decision-making processes and associated activities within households [90]. Parental education significantly contributes to children's higher grades, minor behavioural complications, fewer problems with substance misconduct, improved mental health and increased social awareness [91]. It seems reasonable to claim that well educated individuals in a household have broader control over other members, and in their decision making.

In this study, SES includes gender, age, education, income, marital status, family status, household members and house tenure. Building characteristics comprise the number of bedrooms and house value. Personality traits include agreeableness, consciousness, emotional stability, extraversion, and openness. Table 1 presents the respondents' profiles, including the selected variables' levels, descriptive analysis, percentage of observations, mean, and standard deviation (SD). Age is aggregated into six levels to account for its non-linear effect. Gender is a binary variable coded as 1 for females and 0 for males. Education is aggregated into five levels, while marital status is aggregated into six levels. Family status and house tenure are represented as four levels. Home type was represented as flat, separate, and non-private. The study included income as a piecewise linear variable, categorised into two groups: income up to 150k and income above 150k. House value, bedrooms, personality traits and EC are continuous variables. The correlation between the specific variables utilized to derive the results of this study can be found in Table 5, located in the appendices.

**Table 1:** Profile of the respondents.

Categories	Levels	Percentage	Mean	SD
Age	15 to 24	9.18	0.092	0.289
	25 to 34	20.84	0.208	0.406
	35 to 44	15.63	0.156	0.363
	45 to 54	15.70	0.157	0.364
	55 to 64	15.60	0.156	0.363
	65 & above	23.05	0.231	0.421
Gender	Male	45.21	0.452	0.498
	Female	54.46	0.545	0.498
Education	Bachelor & above	32.74	0.327	0.469
	Diploma	11.99	0.120	0.325
	Certificate	27.24	0.272	0.445
	Year 12	10.69	0.107	0.309
	Y11 & below	17.31	0.173	0.378
Marital Status	Married	39.80	0.398	0.490
	Separated	4.67	0.047	0.211
	Divorced	13.28	0.133	0.339
	Widowed	7.80	0.078	0.268
	In-relation	11.83	0.118	0.323
	In-single	22.61	0.226	0.418
Family Status	Single family	41.39	0.414	0.493
	Family with Kids	26.04	0.260	0.439
	Lone person family	31.42	0.314	0.464
	Non-family members	1.15	0.012	0.107
House Tenure	Owner	59.24	0.593	0.491
	Renter	37.20	0.372	0.483
	Rent-buy	0.04	0.000	0.021
	Free	3.39	0.034	0.181
House Type	Separate	83.66	0.837	0.370
	Flat	15.32	0.153	0.360
	Non-Private	1.00	0.010	0.099
Income	Income up to 150k	82.58	56835.80	44061.20
	Income above 150k	16.93	31807.75	71322.33
Big-Five Traits	Agreeableness	–	5.433	0.940
	Conscientiousness	–	5.117	1.024
	Emotional stability	–	5.246	1.101
	Extraversion	–	4.394	1.088
	Openness to experience	–	4.249	1.096
Building Attributes	Number of bedrooms	–	3.037	1.039
	House value	–	392819.823	491425.101
			
	HEC	–	1579.818	1192.693

In the data cleaning process, households with missing EC values are excluded from the dataset. The outliers in HEC are determined using Z-score [92] where those observations with a Z-score out of the range of ±3 are excluded from the dataset. Moreover, those households reporting more than 8 bedrooms or an annual income higher than $250,000 are excluded from the dataset. Overall, from the 9741 households, 9024 households are included in this study.

### 3.3 Methodology: structural equation modelling

To study the direct, indirect and total effects of SES, building characteristics, and personality traits on HEC, we develop the structural equation modelling (SEM). SEM is chosen because it has the capability of handling complex real-life problems that cannot be modelled using multiple linear regression [93,94]. As SEM is a standard modelling framework, we do not present the mathematical equations, and would refer readers to Ref. [95] for a detailed discussion. The parameter estimation is conducted using the maximum likelihood estimation method [96]. Maximum likelihood requires a relatively smaller sample size (200–400 samples) for parameter estimation. To evaluate the goodness-of-fit, this study uses the Comparative Fit Index (CFI), Tucker-Lewis Index (TLI), Root Mean Square Error of Approximation (RMSEA), Standardized Root Mean Squared Residual (SRMR) and $\chi^{2}/df$ value. The analysis is conducted using Lavaan 0.6–9 in R. Full Information Maximum-likelihood of Lavaan can treat missing values of independent variables by considering missing at random or missing completely at random.

**Fig. 1:**
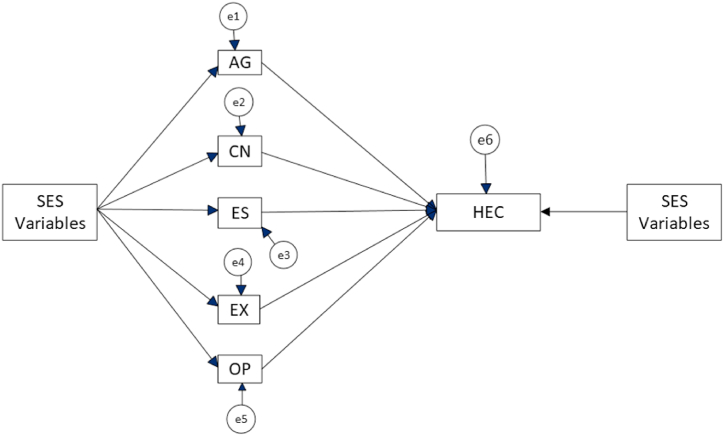
The causal relationship of big-five traits, socioeconomic variables, and household energy consumption. Note. e1-e6 represents error variances. AG = agreeableness, CN = conscientiousness, ES = emotional stability, EX = extraversion, and OP = openness to experience.

To examine the performance of the big-two in explaining HEC, this study designs two models: 1) the big-five model, and 2) the big-two model. In the big-five model, personality traits are observed exogenous variables directly connected to HEC, mediating the effects of SES on HEC. In addition, SES variables are common causal indicators of big-five traits and HEC. The proposed causal relationship of big-five traits, SES variables and HEC is shown Fig. 1.

**Fig. 2:**
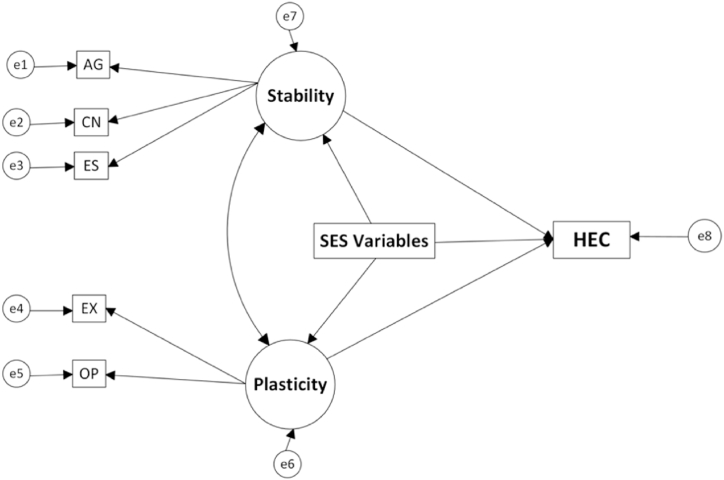
The causal relationship of big-two traits, socioeconomic variables and household energy consumption. Note. e1-e8 represents error variances. AG = agreeableness, CN = conscientiousness, ES = emotional stability, EX = extraversion, and OP = openness to experience. Stability and Plasticity are two latent constructs and big-five traits are effects indicators.

In the big-two model, the observed values of the big-five personality traits are measurement indicators of stability and plasticity. Agreeableness, Conscientiousness and Emotional Stability are indicators of Stability. Extraversion and Openness to experience are two indicators of Plasticity. Along with the direct effects, stability and plasticity mediate the effects of SES on HEC. These two latent variables are then directly connected to HEC. The proposed causal relationship of big-two traits, SES variables and HEC is shown in Fig. 2.

## 4 Analysis and results

As noted in section 3.3, two SEM models are developed to investigate if introducing plasticity and stability will improve the interpretability and prediction accuracy of the model. In this section, first the goodness-of-fit of the two models is compared in section 4.1, and then the relationship between the variables in explaining HEC is discussed in section 4.2.

### 4.1 Goodness-of-fit

The two models are compared using goodness-of-fit indices, i.e., $\chi^{2},R^{2}\text{,}$ CFI, TLI, RMSEA, SRMR, AIC, and BIC value, see Table 2. The discrepancy $\chi^{2}$ is a non-robust fit indicator as it depends on sample size, and is more likely to be significant for large sample sizes [25,35]. In both models, $\chi^{2}$ values are significant (p<0.05). The fit indices (i.e., CFI, TLI, RMSEA, and SRMR) which are insensitive to the sample size are used along with $\chi^{2}$. CFI, ranging from 0 to 1, quantifies the proportional improvement in SEM fit over a null model [97,97]. The threshold of CFI is multi-levels [98]: Poor:CFI≤0.882, Mediocre:0.882≤CFI≤0.904, Fair: 0.904≤CFI≤0.935, Close: 0.935≤CFI≤0.983, and Excellent: CFI≥0.983. No absolute cut points exist. For the big-two and big-five models, CFI is 0.89 and 0.87, respectively, indicating 89% and 87% improvement over the null model. The big-two model fits better than the big-five model. TLI ranges from 0 to 1 and its threshold for acceptable model fit is 0.90 [99] indicating the model accounts for 90% of observed covariance.

**Table 2:** Structural equation model fitting indices of the big-two traits and big-five traits models.

Model	Sample	df	$\frac{\chi^{2}}{\text{df}}\left(p\;\text{value}\right)$	CFI	TLI	AIC	BIC	RMSEA	SRMR	$R^{2}$
Big-Two Traits Model	9024	55	16.20 (0.00)	0.89	0.79	129933	130201	0.041	0.021	0.187
Big-Five Traits Model	9024	69	57.36 (0.00)	0.87	0.24	130227	130612	0.079	0.022	0.147

Note: df: degree of freedom, $\chi^{2}$: test statistic, CFI: comparative fit indices, TLI: Tucker-Lewis Index, RMSEA: root mean square error of approximation, SRMR: standardized root mean square residual.

RMSEA, ranging from 0 to 1, is a measure of poorer fit. RMSEA threshold levels are multi-level [98]: Poor:≥0.114, Mediocre=0.095−0.114, Fair=0.067−0.095, Close: 0.033−0.067, and Excellent: ≤0.033. No absolute cut points exist. This measure is 0.041 (p >0.05) for the big two model and 0.079 (p <0.05) for the big five model. “The SRMR is the absolute mean of all differences between the observed and the model implied correlations” [100] and ranges from 0 to 1. The threshold of SRMR for the acceptable model fit is <0.10 [101]. SRMR is 0.021 for the big-two model and 0.022 for the big-five model. Additionally, $R^{2}$ is a measure of variance explained by the developed model. The big-two model explains 18.7% variance of HEC, while the big-five model explains 14.7% variance.

**Fig. 3:**
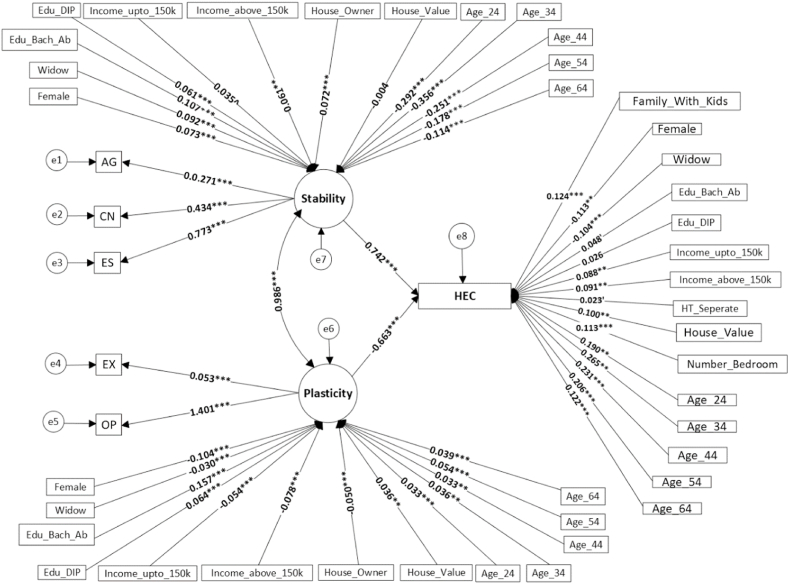
Structural equation modelling results with big-two traits. Standardized solution. AG = agreeableness, CN = conscientiousness, ES = emotional stability, EX = extraversion, and OP = openness to experience. Note. ***P = 0, **P < 0.001, *P < 0.01, ′*P < 0.05, ^P < 0.10*.

**Fig. 4:**
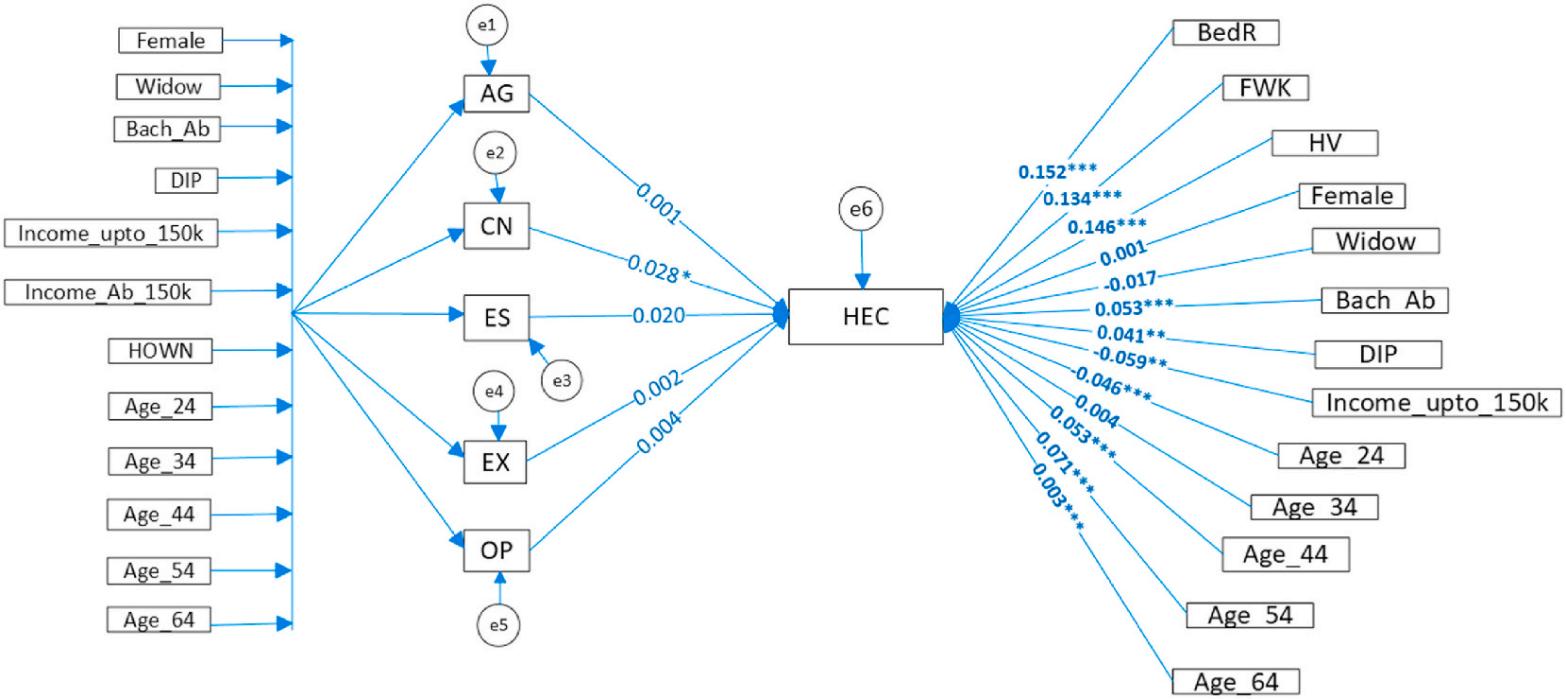
Structural equation modelling results with big-five traits. AG = agreeableness, CN = conscientiousness, ES = emotional stability, EX = extraversion, and OP = openness to experience. Note. ***P = 0, **P < 0.001, *P < 0.01, ′*P < 0.05, ^P < 0.10*.

The big-two model outperforms the big-five model (appendices: Fig. 4), indicating mediating for Stability and Plasticity increases predictability and interpretability. Thus, the study continues with the big-two model (Fig. 3) in the remaining sections.

### 4.2 Parameter estimates

The unstandardised and standardised parameter estimates for big-two and big-five models are presented in the appendix in Tables 6 and 7, respectively. The discussions are focused on the big-two model, where age, education, marital status, family status, household size, house value, income, stability, and plasticity predict HEC. The age level, “65 and above”, is considered as the base case and the remaining levels are used as binary variables. For education, “bachelor and above”, and “diploma” are included as binary variables and the remaining are set as the base case. Widow is coded as 1 and remaining levels of marital status are coded as 0 because it was the only level showing a significant relationship with HEC. For house tenure, “owner” is included as a binary variable, and the remaining levels are set to zero as the base case. “Separate” is coded as 1 and the remaining are coded as 0. For family structure, family with kids is binary, and others are base case.

According to the parameter estimates, five traits significantly load on stability and plasticity, as shown in Fig. 3 and Table 6 (see in appendices). Openness and extraversion's factor loadings on plasticity are positive, implying higher openness and extraversion relate to greater plasticity. Agreeableness, conscientiousness, and emotional stability's factor loadings on stability are also significant and positive, indicating higher levels of these traits imply greater stability. The big-two traits show significant effects on HEC. Stability is positively correlated with HEC [β=0.742,p<0.001], indicating energy-intensive behaviours. Conversely, plasticity is negatively associated with HEC [β=−0.663,p<0.001], indicating energy-saving behaviours.

### 4.3 Direct and indirect effects

The standardised direct, indirect, and total effects are presented in Table 3. Standardised effects provide unitless estimates of coefficients and are scaled based on the variability of the sample or population [102]. Standardised parameters are transformed into unstandardised estimates that eliminate scaling information and allow for informal comparisons of model parameters [103]. The standardised estimates are compared in terms of SD. For example, the estimated direct effect of 0.742 for stability means if stability increases by one SD, HEC will increase by 0.742 SD. Stability and Plasticity have only direct effects but at the same time they mediate the effects of SES. This means SES can affect HEC directly as well as indirectly and through the two latent variables. Indirect effects of SES through Plasticity and Stability are shown in columns 3 & 4, respectively. The indirect effects are calculated as the multiplication of standardised factor loadings from SES to the latent variables into the standardised factor loading from the latent variables to HEC. Total effects (column 5) are the sum of direct and indirect effects.

**Table 3:** Standardised direct, indirect, and total effects in big-two model.

Variables	Direct Effects	Indirect effects of SES Via		Total Effects
		Stability	Plasticity	
**Personal Level**				
Stability	**0.742**	–	–	0.742
Plasticity	**−0.663**	–	–	−0.663
Age 15 to 24	**0.190**	−0.217	−0.022	−0.049
Age 25 to 34	**0.265**	−0.264	−0.024	−0.023
Age 35 to 44	**0.231**	−0.186	−0.022	0.023
Age 45 to 54	**0.206**	−0.132	−0.036	0.038
Age 55 to 64	**0.122**	−0.085	−0.026	0.012
Female	**−0.113**	0.054	0.069	0.010
Widow	−0.104	0.068	0.020	−0.016
Bachelor and above	**0.048**	0.079	−0.104	0.023
Diploma	0.026	0.045	−0.042	0.029
**Household Level**				
Income up to 150k	**0.088**	0.026	0.036	0.150
Income above 150k	**0.091**	0.045	0.052	0.188
House value	0.100	−0.003	−0.024	0.073
House owner	–	0.053	0.033	0.087
Number of bedrooms	0.113	–	–	0.113
Separate house	**0.023**	–	–	0.023
Family with Kids	**0.124**	–	–	0.124

## 5 Discussions

The comparison between the big-two and big-five models provides further evidence in support of the hypothesis that stability and plasticity traits can better explain EC. The parameter estimates presented in section 4.2 demonstrate that all established connections in the model are statistically significant. This section delves into the behavioural interpretations of the model by carefully examining and contrasting the outcomes with the findings from previous studies reported in the literature.

### 5.1 Impacts of big-two traits on EC

This study investigates HEC using a representative sample that includes household and individual attributes. The household attributes include EC, income, house tenure, house value, number of bedrooms, and family status. The personality traits, age, education, gender, and marital status are individual attributes of representative.

Stability shows a positive relationship with HEC; a one SD increase in stability leads to a 0.742 SD increase in HEC, while all other variables remain unchanged, see Table 3. The stability trait poses some inherent characteristics, e.g., lack of creativity, avoiding risks, and lower adaptability to novel situations. This trait is understood as the process by which cybernetic systems care for goal-directedness when facing disruptive impulses [39] and abstaining from a range of behaviours linked to disruptive impulse is related to stability [59]. Stable people lack the ability to explore and reframe ideas to adapt to novel situations rather than rely on society's desire [35]. These behaviours reduce the tendency to adapt to changing circumstances and adjust consumption habits. Previous studies find that stability is negatively associated with creativity [30,75], social network use [74], and anxiety and stress [30]. These people dislike changes that push them to go beyond their comfort zone [74]. Energy curtailment may reduce living comfort [19], which requires a change in consumption habits and lifestyle [104]. Individuals with higher stability resist adopting energy-saving behaviours therefore showing higher HEC and greenhouse gas emissions.

According to the modelling results, the positive correlation between stability and HEC can be explained by their adverse sustainable behaviour, as people with higher stability traits tend to be more rule-bound and conformist [35]. Consequently, they may engage in energy-consuming activities that result in higher energy consumption levels. For instance, highly conscientious individuals may adhere to household routines [27] including excessive use of energy-consuming appliances like heating and cooling systems, lighting, and electronic devices. Similarly, highly agreeable individuals may prioritise social harmony and be more likely to engage in energy-consuming activities to accommodate the needs or preferences of others, such as using energy to maintain a comfortable temperature for their family or guests, even if it results in higher HEC. Thus, policymakers can target individuals with stability traits to capture their energy consumption behaviour when developing environmentally sustainable policies to meet the NZE target.

Plasticity shows a negative relationship with HEC. According to modelling results in Table 3, one SD increase in plasticity while all other variables remain unchanged is associated with a 0.663 SD decrease in HEC. Such a relationship of plasticity is attributed to some inherent characteristics such as exploration, flexibility, higher capacity to adjust to unfamiliar states, challenging social norms, and seeking out stimulating experiences [35,37]. Previous studies show that plasticity is positively associated with creativity [30,75] and social network use [74]. The plasticity meta-trait can be understood as the process by which cybernetic systems maintain and examine the unknown, either cognitively (i.e., openness) or behaviourally (i.e., extraversion) [39]. The energy-saving behaviour observed in people with higher plasticity can be explained by their higher “acceptance to changing contexts”. People with higher plasticity levels are more likely to explore different lifestyles and EC habits to adjust their behaviour in response to environmental issues.

The identified relationship between plasticity and HEC can be explained in terms of sustainable behaviour because individuals with high plasticity are more likely to be interested in environmental issues and to adopt environmentally friendly behaviours [35,37]. They are also more likely to be receptive to new information and innovative solutions for reducing EC. Therefore, individuals with high levels of plasticity are more likely to adopt sustainable behaviours, including reducing HEC. They are more likely to be motivated by environmental concerns and take action to reduce their carbon footprint. Furthermore, adopting sustainable behaviours such as lower HEC consumption can lead to a range of positive outcomes, including lower energy bills, improved air quality, and reduced greenhouse gas emissions. Individuals can contribute to a more sustainable future and help mitigate the negative impacts of climate change by reducing their energy consumption. The policymaker can enhance the behaviour of plasticity traits to reduce greenhouse gas emissions and environmental degradation in the strategic development of interventions.

### 5.2 The direct effects of sociodemographic attributes on HEC

Moving from lower to higher brackets in Table 3, EC decreases with age when other variables remain constant. The finding is consistent with recent studies [18,105], though the effects may vary based on culture and geography. Income and HEC exhibit a piecewise linear relationship. Increasing income by one SD is associated with a corresponding increase in HEC by 0.088 and 0.091 SDs for households with income up to 150k and above 150k, respectively. This outcome is consistent with past research [[106], [107], [108], [109]]. Low-income individuals have lower HEC as they prioritise reducing living costs by limiting EC [19,110], though they cannot afford energy-efficient appliances. Higher-income individuals tend to invest in energy-saving appliances but paradoxically consume more energy with such appliances to maintain comfort [19,111,112]. The standardised direct effect for those with “bachelor and above” on HEC is 0.048. Accordingly, higher educational attainments are associated with increased HEC and are consistent with recent studies [110,113,114].

The direct effect for females is −0.113, which shows a decreasing effect on HEC. The finding supports the notion that females exhibit more energy-saving behaviour due to their greater environmental awareness [17,115]. “Families with kids” show a higher HEC, consistent with earlier studies [18,116]. The standard effect of separate house is 0.023 and positive, and past studies [117] reported similar findings. More expensive houses and number of bedrooms are directly related to the households’ size and heating area, which causes higher EC.

### 5.3 The effect of sociodemographic attributes on the big-two personality attributes

The results confirm the correlations of SES variables with personality traits. While the correlation is small in some cases (see Table 4), it is still significant, and can be used to identify target groups for policy interventions. SES variables have opposite effects on stability versus plasticity. A 1-SD increase in income for those with an “income up to 150k” and an “income above 150k” is associated with 0.035 and 0.061 SD increase in stability, respectively. Similarly, a 1-SD increase in “income up to 150k” and “income above 150k” is associated with 0.054 and 0.078 SD decrease in plasticity, respectively. So, higher income leads to more stability but less plasticity. Higher-income people consume more energy with energy-efficient appliances to maintain a comfortable environment, which could explain their increased stability [112]. Females are more stable than males. The direct effects of females on stability and plasticity are 0.073 and −0.104, respectively. Widows show a positive association with stability, possibly due to their reluctance to change based on past unpleasant experiences.

**Table 4:** direct effects of sociodemographic variables on latent stability and plasticity in big-two model.

SES Variables	Stability			Plasticity		
	B	β	P-value	B	β	P-value
Age 15 to 24	−1.123	**−0.292**	0.000	0.116	**0.033**	0.000
Age 25 to 34	−0.973	**−0.356**	0.000	0.091	**0.036**	0.001
Age 35 to 44	−0.767	**−0.251**	0.000	0.094	**0.033**	0.001
Age 45 to 54	−0.542	**−0.178**	0.000	0.152	**0.054**	0.000
Age 55 to 64	−0.349	**−0.114**	0.000	0.109	**0.039**	0.000
Female	0.162	**0.073**	0.000	−0.213	**−0.104**	0.000
Widow	0.381	**0.092**	0.000	−0.116	**−0.030**	0.000
Bachelor and above	0.253	**0.107**	0.000	0.340	**0.157**	0.000
Diploma	0.210	**0.061**	0.000	0.201	**0.064**	0.000
Income upto 150k	0.039	**0.035**	0.060	−0.056	**−0.054**	0.000
Income above 150k	0.066	**0.061**	0.002	−0.078	**−0.078**	0.000
House value	−0.005	**−0.004**	0.830	0.043	**0.036**	0.001
House owner	0.163	**0.072**	0.000	−0.104	**−0.050**	0.000

Note: B: unstandardised coefficient; β:standardizedcoefficient.

Homeownership is positively associated with stability and negatively associated with plasticity, with estimated standardised effects of 0.072 and −0.050, respectively. House value shows a positive effect on plasticity and is insignificant in stability. Stability increases with age, while plasticity increases until age 54 with a minimal increase. The non-linear effects of age levels are more profound for stability than plasticity. Education is positively associated with stability and plasticity. That means those with higher educational attainments show higher levels of stability and plasticity. Nevertheless, the effect on plasticity is larger. A bachelor's degree or higher has a direct effect of 0.107 on stability and 0.157 on plasticity. For a “diploma”, the effects on stability and plasticity are 0.061 and 0.064, respectively.

### 5.4 Policy implications

This study supports targeted policies to promote energy-saving behaviours. Big-two traits, which should be considered in energy-saving interventions, play a vital role in determining EC behaviour and responsiveness to policies. Those with higher plasticity are more likely to adjust EC behaviour. Therefore, by targeting individuals with higher plasticity, energy-saving campaigns and policies can increase the likelihood of success. The behavioural interventions e.g., green personality [79] and lifestyle change [12] are shown to be effective strategies, and promoting these interventions among individuals with higher plasticity facilitates their widespread. Meanwhile, those with higher stability are less likely to adjust their behaviour in response to a new policy, therefore policies targeting this cohort would need to offer higher incentives to reduce EC. Policymakers can use this insight to design specific policies for individuals with different stability and plasticity levels to account for energy-intensive and energy-saving behaviours.

The challenge of targeting individuals with specific personality traits in policy-making is addressed by this study, which suggests that stability and plasticity traits are significantly linked to measurable demographic attributes. Therefore, these attributes can be used to infer individuals’ personality traits. For example, older people and high-income individuals are more likely to exhibit higher stability traits, which suggests that energy-saving programs may be required to adjust EC behaviour in these individuals. Similarly, females and house owners are positively associated with stability, where house owners may adopt energy-efficient technologies during construction, but they are more prone not to cut their comfort and consume more energy with energy-efficient appliances. However, energy efficient appliances can reduce around 13.97% of household EC [118].

This study identifies the relationship between socioeconomic variables (i.e., age, gender, marital status, education, income and house ownership) and big-two personality traits. It shows a distinct relationship with big-two traits, that can help in community energy saving practices. Individuals who score high on the plasticity trait show lower HEC, energy-saving behaviour. The plasticity trait encompasses curiosity, adaptability to new situations, flexibility in thinking, challenging societal norms, seeking stimulating experiences, and embracing positive emotions [30]. Individuals who score high on plasticity traits are more likely to change their behaviour towards new and sustainable options. Capitalising on the inherent characteristics of these individuals, policy interventions can effectively promote usages of households’ energy-saving appliances. Education and awareness campaigns can play a pivotal role in encouraging them to embrace sustainable energy-saving alternatives, i.e., the use of solar power.

Individuals with higher stability traits tend to have limited adaptability, preferring to avoid distress, uncertainty, and hostility while displaying a fear of the unknown and aversion to novelty [30,35]. Compared to those with high plasticity, these individuals require stronger incentives. Policymakers could focus on providing incentives to encourage them to lower HEC. These incentives could include tax deductions, variable energy pricing to different thresholds, rebates on smart households’ devices, energy-efficient appliances, solar panels, and insulation. By highlighting the benefits of environmental sustainability options, i.e., cost savings, improved health effects, and reduced environmental impact, policymakers can provide crucial information that motivates lower HEC. To encourage them, community engagement is an important measure to involve them in developing eco-friendly and energy-saving community services considering their perspectives and preferences. Regular feedback as policy dialogue from these individuals is vital to tailor the services to their unique needs. They can be actively involved in sustainable community development, which may influence them to lower their self-consumption to be environmentally friendly.

This study provides guidance for policymakers to intervene in HEC behaviour based on meta-traits. Results reveal that energy-saving interventions need to consider individual differences, importantly stability and plasticity, to improve the effectiveness of policies, by identifying individuals more likely to change and those who needs to be incentivised.

## 6 Conclusions

This study explored the effects of stability and plasticity traits on HEC by developing two structural equation models, including a big-two traits model and a big-five traits model (benchmark). The models were calibrated using HILDA data and compared based on goodness-of-fit measures (i.e., $\chi^{2}$, CFI, TLI, RMSEA, SRMR, AIC, BIC, and $R^{2}$). The parameters of the models were estimated using maximum likelihood estimation. The big-two model performed better than the benchmark model and offered more behavioural interpretability, including capturing the link between personality traits and sociodemographic attributes. This feature is essential in identifying target groups for policy interventions.

The results confirmed that the big-two traits were significantly associated with HEC. Stability was positively associated with HEC, indicating that those with higher stability used more energy in their homes, while plasticity had a negative correlation, suggesting that individuals with higher plasticity scores were more likely to reduce energy use. Stability increased with the increase in the age of respondents. SES significantly moderated stability and plasticity in the process of HEC behaviour. Income and gender were significant moderators of stability and plasticity, with higher income associated with increased stability and decreased plasticity. Females were found to be more stable than males. Homeownership was associated with energy-intensive behaviour and lower plasticity. Therefore, it is reasonable to claim that the sociodemographic attributes are effective tools to describe peoples’ personality traits.

The study suggests that policymakers can devise more effective interventions for HEC by targeting stability and plasticity traits. The results of this study can help policymakers in tailoring targeted interventions focusing on sustainable societies. This study's modelling framework included various socioeconomic and demographic status attributes, but could include more, such as occupation and employment status. It only considered one individual from each household, although EC is a group decision. Future research should analyse group decision-making dynamics and cultural background to capture the broader effects of personality and demographic status on HEC. Self-reported personality trait data were used; future research could compare self-reported and informant-reported data. To advance the understanding of HEC dynamics, the incorporation of big-two personality traits represents a crucial and innovative dimension. This investigation pioneers the exploration of stability and plasticity traits in the context of HEC behaviour, opening avenues for extensive research in the field. In the context of big-two traits, the temporal aspects of energy-saving, the influence of life events, and the dynamics of household decision-making are fertile grounds for investigation. The recommended longitudinal studies promise to unravel the intricate relationship between stability and plasticity traits and changes in energy-saving practices over time. Furthermore, the potential impact of targeted behavioural interventions designed to align with specific personality traits and the implications for tailoring energy-related policies based on these traits underscore the practical relevance for further research.

## Ethics approval

The HILDA dataset used in this study was collected from the Australian Data Archives (ADA) with the approval of ADA for this study. The modelling framework was presented to the Human Research Ethics Committee (HREC) at the University of New South Wales (UNSW), and it was approved by the HREC before conducting the study.

## Data availability statement

This research relies on the restricted release of Wave-2017 of HILDA dataset. This particular release is exclusively accessible to researchers within Australia who have obtained prior authorization from ADA (Australian Data Archive). It's essential to note that this restricted version of the HILDA dataset is not publicly accessible. Consequently, the authors are unable to provide access to the dataset utilized in this study for reporting its outcomes. For researchers interested in exploring energy consumption utilizing the same dataset, they can pursue access by submitting an approval request to ADA through the “Household, Income and Labour Dynamics in Australia Dataverse” link.

## CRediT authorship contribution statement

**Md Shahin:** Writing – original draft, Visualization, Validation, Methodology, Investigation, Formal analysis, Data curation. **Milad Ghasri:** Writing – review & editing, Validation, Supervision, Resources, Methodology, Formal analysis, Conceptualization. **Alireza Abbasi:** Writing – review & editing, Supervision, Methodology, Conceptualization.

## Declaration of competing interest

The authors declare that they have no known competing financial interests or personal relationships that could have appeared to influence the work reported in this paper.

## Figures and Tables

**Table 5:** Correlation of variables used in SEM models.

		1	2	3	4	5	6	7	8	9	10	11	12	13	14	15	16	17	18	19	20	21
1	Conscientiousness	–	–	–	–	–	–	–	–	–	–	–	–	–	–	–	–	–	–	–	–	–
2	Agreeableness	0.28	–	–	–	–	–	–	–	–	–	–	–	–	–	–	–	–	–	–	–	–
3	Emotional stability	0.34	0.12	–	–	–	–	–	–	–	–	–	–	–	–	–	–	–	–	–	–	–
4	Openness to experience	0.07	0.31	−0.22	–	–	–	–	–	–	–	–	–	–	–	–	–	–	–	–	–	–
5	Extraversion	0.15	0.19	0.18	0.07	–	–	–	–	–	–	–	–	–	–	–	–	–	–	–	–	–
6	Age 15 to 24	−0.09	−0.06	−0.14	0.01	0.04	–	–	–	–	–	–	–	–	–	–	–	–	–	–	–	–
7	Age 25 to 34	−0.05	−0.03	−0.16	0.03	0.03	−0.15	–	–	–	–	–	–	–	–	–	–	–	–	–	–	–
8	Age 35 to 44	−0.03	0.01	−0.07	0.03	−0.01	−0.13	−0.22	–	–	–	–	–	–	–	–	–	–	–	–	–	–
9	Age 45 to 54	0.01	0.03	−0.01	0.05	−0.02	−0.13	−0.22	−0.19	–	–	–	–	–	–	–	–	–	–	–	–	–
10	Age 55 to 64	0.03	0.03	0.06	0.01	−0.01	−0.13	−0.22	−0.19	−0.19	–	–	–	–	–	–	–	–	–	–	–	–
11	Female	0.08	0.26	0.02	−0.03	0.11	0.01	0.04	0.02	−0.01	−0.01	–	–	–	–	–	–	–	–	–	–	–
12	Widow	0.05	0.04	0.17	−0.11	−0.01	−0.09	−0.14	−0.11	−0.10	−0.04	0.14	–	–	–	–	–	–	–	–	–	–
13	Bachelor and above	0.09	0.06	0.03	0.20	0.01	−0.07	0.10	0.09	0.02	−0.02	0.06	−0.12	–	–	–	–	–	–	–	–	–
14	Diploma	0.02	0.03	0.03	0.03	0.01	−0.04	−0.01	0.01	0.01	0.02	0.04	−0.03	−0.27	–	–	–	–	–	–	–	–
15	Income up to 150k	0.02	0.00	−0.06	0.03	0.01	−0.02	0.09	0.08	0.02	0.01	−0.02	−0.14	0.03	0.03	–	–	–	–	–	–	–
16	Income above 150k	0.06	0.02	0.02	0.02	0.03	−0.08	0.03	0.10	0.11	0.02	−0.01	−0.11	0.27	0.01	−0.59	–	–	–	–	–	–
17	House value	0.12	0.04	0.13	0.03	0.01	−0.17	−0.18	0.00	0.05	0.10	−0.03	0.03	0.22	0.03	−0.02	0.24	–	–	–	–	–
18	House owner	0.13	0.04	0.17	−0.05	−0.02	−0.24	−0.22	−0.01	0.06	0.13	−0.03	0.09	0.11	0.05	0.02	0.18	0.65	–	–	–	–
19	Number of bedrooms	0.05	0.02	0.02	−0.01	0.02	−0.08	−0.06	0.10	0.07	0.04	−0.02	−0.09	0.07	0.04	0.09	0.19	0.34	0.33	–	–	–
20	Separate house	0.00	0.01	0.02	−0.03	−0.02	−0.09	−0.05	0.04	0.05	0.03	−0.02	−0.03	−0.03	0.04	0.07	0.06	0.20	0.29	0.49	–	–
21	Family with Kids	−0.05	−0.01	−0.12	−0.03	0.03	−0.07	0.20	0.38	0.04	−0.21	0.09	−0.15	0.08	0.00	0.15	0.11	0.00	−0.03	0.23	0.14	–

**Table 6:** Structural equation modelling results of big-two model.

Measurement Model							
Variables	Β	Standard Error	z-value	P-value	β	ci.lower	ci.upper
Stability = ∼							
Consciousness CN	0.392	0.014	27.140	0.000	0.434	0.402	0.466
Agreeableness AG	0.245	0.028	8.825	0.000	0.271	0.212	0.331
Emotional Stability ES	0.703	0.027	26.085	0.000	0.773	0.726	0.821
Plasticity = ∼							
Openness OP	1.388	0.165	8.423	0.000	1.401	1.089	1.713
Extraversion EX	0.052	0.009	5.868	0.000	0.053	0.035	0.071
**Structural Model**							
Stability ∼							
Age 15 to 24	−1.123	0.070	−16.002	0.000	−0.292	−0.325	−0.259
Age 25 to 34	−0.973	0.057	−16.956	0.000	−0.356	−0.394	−0.319
Age 35 to 44	−0.767	0.058	−13.299	0.000	−0.251	−0.286	−0.216
Age 45 to 54	−0.542	0.053	−10.144	0.000	−0.178	−0.211	−0.144
Age 55 to 64	−0.349	0.049	−7.061	0.000	−0.114	−0.145	−0.083
Income upto 150k	0.039	0.021	1.882	0.060	0.035	−0.001	0.070
Income above 150k	0.066	0.021	3.129	0.002	0.061	0.023	0.099
Bachelor and above	0.253	0.035	7.176	0.000	0.107	0.078	0.136
Diploma	0.210	0.045	4.634	0.000	0.061	0.036	0.087
Female	0.162	0.038	4.247	0.000	0.073	0.039	0.106
Widow	0.381	0.060	6.362	0.000	0.092	0.064	0.120
House owner	0.163	0.040	4.034	0.000	0.072	0.037	0.107
House value	−0.005	0.022	−0.215	0.830	−0.004	−0.037	0.030
Plasticity ∼							
Age 15 to 24	0.116	0.033	3.572	0.000	0.033	0.015	0.051
Age 25 to 34	0.091	0.027	3.441	0.001	0.036	0.016	0.057
Age 35 to 44	0.094	0.027	3.441	0.001	0.033	0.014	0.052
Age 45 to 54	0.152	0.029	5.256	0.000	0.054	0.034	0.075
Age 55 to 64	0.109	0.026	4.129	0.000	0.039	0.021	0.057
Income upto 150k	−0.056	0.012	−4.733	0.000	−0.054	−0.077	−0.032
Income above 150k	−0.078	0.013	−5.849	0.000	−0.078	−0.104	−0.052
Bachelor and above	0.340	0.045	7.547	0.000	0.157	0.117	0.196
Diploma	0.201	0.034	5.915	0.000	0.064	0.043	0.085
Female	−0.213	0.028	−7.586	0.000	−0.104	−0.13	−0.078
Widow	−0.116	0.032	−3.596	0.000	−0.030	−0.047	−0.014
House owner	−0.104	0.023	−4.499	0.000	−0.050	−0.072	−0.029
House value	0.043	0.012	3.474	0.001	0.036	0.016	0.057
HEC ∼							
Stability	0.532	0.126	4.234	0.000	0.742	0.400	1.085
Plasticity	−0.518	0.126	−4.116	0.000	−0.663	−0.978	−0.348
Age 15 to 24	0.523	0.174	3.001	0.003	0.190	0.066	0.314
Age 25 to 34	0.519	0.149	3.476	0.001	0.265	0.116	0.414
Age 35 to 44	0.504	0.123	4.097	0.000	0.231	0.120	0.341
Age 45 to 54	0.449	0.101	4.444	0.000	0.206	0.115	0.296
Age 55 to 64	0.268	0.072	3.708	0.000	0.122	0.058	0.187
Female	−0.180	0.056	−3.217	0.001	−0.113	−0.181	−0.044
Widow	−0.310	0.081	−3.853	0.000	−0.104	−0.158	−0.051
Bachelor and above	0.081	0.037	2.221	0.026	0.048	0.006	0.090
Diploma	0.064	0.039	1.631	0.103	0.026	−0.005	0.058
Income upto 150k	0.072	0.022	3.234	0.001	0.088	0.035	0.142
Income above 150k	0.070	0.027	2.646	0.008	0.091	0.024	0.158
House value	0.093	0.016	5.730	0.000	0.100	0.066	0.134
Number of bedrooms	0.093	0.010	9.363	0.000	0.113	0.089	0.136
Separate house	0.048	0.024	1.988	0.047	0.023	0.000	0.045
Family with kids	0.224	0.021	10.579	0.000	0.124	0.101	0.146

Note: B: unstandardised coefficient, β:*standardised coefficient, ci: confidence interval (standardised)*.

**Table 7:** Structural equation modelling results big-five traits model.

Variables	Β	Standard Error	z-value	P-Value	β	ci.lower	ci.upper
Agreeableness ∼							
Age 15 to 24	−0.263	0.047	−5.566	0.000	−0.076	−0.103	−0.049
Age 25 to 34	−0.176	0.038	−4.580	0.000	−0.071	−0.102	−0.041
Age 35 to 44	−0.068	0.039	−1.719	0.086	−0.025	−0.053	0.003
Age 45 to 54	−0.013	0.038	−0.344	0.731	−0.005	−0.032	0.022
Age 55 to 64	−0.006	0.037	−0.169	0.866	−0.002	−0.028	0.024
Income above 150k	0.055	0.032	1.711	0.087	0.025	−0.006	0.052
Income upto 150k	0.013	0.013	0.995	0.32	0.013	−0.017	0.036
Bachelor and above	0.108	0.025	4.256	0.000	0.051	0.028	0.074
Diploma	0.096	0.034	2.812	0.005	0.031	0.009	0.053
Female	0.520	0.022	23.785	0.000	0.259	0.239	0.280
Widow	−0.040	0.046	−0.875	0.382	−0.011	−0.034	0.014
House Owner	−0.001	0.025	−0.028	0.977	0.000	−0.025	0.024
Conscientiousness ∼							
Age 15 to 24	−0.403	0.048	−8.380	0.000	−0.116	−0.144	−0.089
Age 25 to 34	−0.300	0.039	−7.684	0.000	−0.122	−0.153	−0.091
Age 35 to 44	−0.306	0.040	−7.588	0.000	−0.111	−0.140	−0.083
Age 45 to 54	−0.207	0.039	−5.312	0.000	−0.075	−0.103	−0.048
Age 55 to 64	−0.139	0.037	−3.729	0.000	−0.051	−0.077	−0.024
Income above 150k	0.205	0.033	6.205	0.000	0.093	0.062	0.121
Income upto 150k	0.018	0.013	1.380	0.167	0.019	−0.011	0.043
Bachelor and above	0.150	0.026	5.785	0.000	0.070	0.047	0.094
Diploma	0.099	0.035	2.823	0.005	0.032	0.010	0.054
Female	0.164	0.022	7.359	0.000	0.082	0.060	0.103
Widow	0.032	0.047	0.676	0.499	0.008	−0.016	0.034
House Owner	0.129	0.026	5.031	0.000	0.063	0.039	0.088
Emotional Stability ∼							
Age 15 to 24	−0.766	0.047	−16.333	0.000	−0.219	−0.245	−0.194
Age 25 to 34	−0.707	0.038	−18.552	0.000	−0.284	−0.314	−0.255
Age 35 to 44	−0.583	0.039	−14.859	0.000	−0.210	−0.237	−0.183
Age 45 to 54	−0.431	0.038	−11.336	0.000	−0.156	−0.182	−0.129
Age 55 to 64	−0.276	0.036	−7.577	0.000	−0.099	−0.125	−0.074
Income above 150k	0.135	0.032	4.195	0.000	0.061	0.033	0.089
Income upto 150k	0.044	0.013	3.376	0.001	0.044	0.018	0.070
Bachelor and above	0.135	0.025	5.355	0.000	0.063	0.040	0.086
Diploma	0.131	0.034	3.850	0.000	0.042	0.020	0.063
Female	0.037	0.022	1.708	0.088	0.018	−0.003	0.039
Widow	0.285	0.046	6.226	0.000	0.075	0.052	0.099
House Owner	0.101	0.025	4.059	0.000	0.049	0.025	0.073
Extraversion ∼							
Age 15 to 24	0.115	0.048	2.367	0.018	0.033	0.006	0.061
Age 25 to 34	0.005	0.039	0.128	0.898	0.002	−0.030	0.033
Age 35 to 44	−0.088	0.041	−2.178	0.029	−0.032	−0.062	−0.003
Age 45 to 54	−0.091	0.039	−2.319	0.020	−0.033	−0.062	−0.005
Age 55 to 64	−0.055	0.038	−1.471	0.141	−0.020	−0.047	0.007
Income above 150k	0.176	0.033	5.313	0.000	0.081	0.050	0.109
Income upto 150k	0.011	0.013	0.848	0.396	0.012	−0.018	0.036
Bachelor and above	−0.048	0.026	−1.846	0.065	−0.023	−0.047	0.001
Diploma	−0.001	0.035	−0.024	0.981	0.000	−0.023	0.022
Female	0.221	0.022	9.860	0.000	0.111	0.089	0.133
Widow	−0.061	0.047	−1.292	0.196	−0.016	−0.041	0.009
House Owner	−0.034	0.026	−1.309	0.191	−0.017	−0.042	0.008
Openness ∼							
Age 15 to 24	0.150	0.048	3.112	0.002	0.043	0.016	0.070
Age 25 to 34	0.138	0.039	3.509	0.000	0.055	0.025	0.086
Age 35 to 44	0.163	0.040	4.044	0.000	0.059	0.030	0.087
Age 45 to 54	0.253	0.039	6.445	0.000	0.091	0.063	0.118
Age 55 to 64	0.177	0.037	4.724	0.000	0.063	0.037	0.090
Income above 150k	−0.190	0.033	−5.744	0.000	−0.085	−0.114	−0.056
Income upto 150k	−0.022	0.013	−1.633	0.102	−0.022	−0.048	0.005
Bachelor and above	0.516	0.026	19.83	0.000	0.239	0.216	0.262
Diploma	0.303	0.035	8.649	0.000	0.097	0.075	0.119
Female	−0.099	0.022	−4.410	0.000	−0.048	−0.070	−0.027
Widow	−0.216	0.047	−4.582	0.000	−0.057	−0.081	−0.033
House Owner	−0.093	0.026	−3.630	0.000	−0.045	−0.070	−0.021
HEC ∼							
Agreeableness	0.000	0.009	0.046	0.964	0.001	−0.023	0.023
Conscientiousness	0.022	0.009	2.444	0.015	0.028	0.005	0.050
Emotional Stability	0.016	0.010	1.628	0.103	0.020	−0.004	0.044
Openness	0.003	0.009	0.346	0.730	0.004	−0.019	0.028
Extraversion	0.002	0.009	0.220	0.826	0.002	−0.019	0.023
House value	0.135	0.010	12.932	0.000	0.146	0.124	0.167
Income upto 150k	−0.046	0.008	−5.410	0.000	−0.059	−0.082	−0.039
Number of bedrooms	0.125	0.009	13.940	0.000	0.152	0.131	0.173
Age 15 to 24	−0.126	0.034	−3.746	0.000	−0.046	−0.069	−0.021
Age 25 to 34	0.007	0.029	0.252	0.801	0.004	−0.025	0.033
Age 35 to 44	0.117	0.031	3.757	0.000	0.053	0.026	0.081
Age 45 to 54	0.155	0.028	5.504	0.000	0.071	0.046	0.096
Age 55 to 64	0.080	0.027	2.993	0.003	0.037	0.013	0.061
Bachelor and above	0.089	0.019	4.735	0.000	0.053	0.031	0.075
Diploma	0.101	0.025	4.006	0.000	0.041	0.021	0.062
Female	0.002	0.017	0.127	0.899	0.001	−0.019	0.022
Widow	−0.050	0.034	−1.478	0.139	−0.017	−0.039	0.006
Family with kids	0.243	0.021	11.419	0.000	0.134	0.111	0.157

Note: B: unstandardised coefficient, β:*standardised coefficient, ci: confidence interval (standardised)*.
